# Assessing the Anxiety and Knowledge about Monkeypox Virus: A Cross-Sectional Cohort Study

**DOI:** 10.7759/cureus.47806

**Published:** 2023-10-27

**Authors:** Omar A Alqthami, Rayan H Alaseeri, Abdullah A Alzahrani, Abdullah A Al-otaibi, Yazan A Alghoraibi, Ahmed S Abdel-Moneim, Imad A A. Mohamed, Ali H S. Alzahrani, AbdulRahman N Al-Ghamdi

**Affiliations:** 1 College of Medicine, Taif University, Taif, SAU; 2 Department of Microbiology, College of Medicine, Taif University, Taif, SAU; 3 Department of Community Medicine, College of Medicine, Taif University, Taif, SAU

**Keywords:** monkeypox epidemic, kingdom of saudi arabia (ksa), health awareness, generalized anxiety disorder (gad), monkeypox virus

## Abstract

Introduction

Recent cases of human monkeypox virus (MPXV) infections have raised global health concerns, as sporadic instances have occurred in various regions, prompting investigations into the potential for increased transmission. This underscores the importance of effective communication strategies in addressing the emerging challenges associated with this viral ailment. The study was conducted to understand public anxiety and knowledge related to MPXV infection, particularly in the context of emerging infectious diseases. Our aims included assessing anxiety levels and knowledge about monkeypox infection among the Saudi population, as well as their willingness to receive vaccinations if available.

Methods

A cross-sectional cohort study among the adult Saudi population was conducted. A questionnaire with four sections, including demographic data and disease knowledge, comprised optimized questions of the standard generalized anxiety disorder assessment (GAD-7) as well as questions related to acceptance of getting the vaccine if it could be afforded.

Results

Out of a total of 5298 participants, 927 (17.5%) showed different degrees of GAD-7 anxiety. Females showed a significantly higher rate of anxiety (487/2189, 22.2%) than males (440/3109, 14.2%). People aged 46 to 55 and >55 years old showed significantly higher rates of anxiety (30.7% and 27.2%). There is an overall decrease in knowledge and awareness about the MPXV. Interestingly, 59% of the participants admitted that they would get the MPXV vaccine if it were made available. There was a positive correlation between the anxiety level and the response of people toward the MPXV vaccine if it were available.

Conclusion

Our study underscores a significant level of anxiety and a notable lack of awareness concerning MPXV infection. Although a substantial number of participants expressed their willingness to receive an MPXV vaccine, our findings emphasize the pressing need for improved public education and awareness campaigns to alleviate anxiety levels and enhance understanding of this infectious disease. This effort is crucial for mitigating health concerns and facilitating well-informed decision-making among the Saudi population.

## Introduction

There is an increase in concerns about a new viral pandemic on the horizon caused by the monkeypox virus (MPXV) after the COVID-19 pandemic [1]. Human MPXV is a double-stranded DNA virus of the Orthopoxvirus genus of the family Poxviridae [2]. The MPXV is not new; it was first discovered in 1958 in Copenhagen. It was isolated from a research facility that housed laboratory animals where monkeys had been shipped from Singapore to Denmark [1]. In 1958, two outbreaks of non-fatal nodular skin eruptions were reported in two shipments of cynomolgus monkeys (*Macaca fascicularis*) shipped from Singapore to Copenhagen. About 51 to 62 days after their arrival, 20% to 30% of them developed a clinical illness. A slow recruitment in cycles of inapparent infections with subsequent emergence of clinical disease in susceptible monkeys was speculated [3]. It was found to be antigenically closely related to the vaccinia-variola subgroup. In 1962, in a primate colony in the USA, the virus appeared to induce inapparent infections in different species and also fatalities in irradiated animals [4]. In that study, the appearance of clinical disease in irradiated cynomolgus monkeys implies that the virus could be reactivated from latency in infected animals.

Monkeys and humans are incidental hosts. The reservoirs are believed to be mainly rodents, involving squirrels and Gambian rats. Animal-to-human transmission of MPXV has been proven to occur through contact with an infected animal's bodily fluid or through a bite. The virus transmits from human to human in either of the following ways: large air droplets with prolonged face-to-face contact, contact with infected skin lesions, bodily fluids, contaminated surfaces, or personal objects. It is also proven to cross the placenta from the mother to the fetus [5].

In August 1970, the first human case of monkeypox was identified in a nine-year-old child with smallpox-like vesicular skin lesions in the village of Bukenda in the Equatorial region of Zaire (now the Democratic Republic of the Congo (DRC)). This patient was discovered during a period of intensified smallpox surveillance conducted nine months after the World Health Organization (WHO) certified the eradication of smallpox in the DRC [6]. Ever since its discovery, the disease has been endemic to Central and West Africa, with intermittent, sporadic cases of monkeypox transmitted from local wildlife reported among humans [2].

Forty-seven cases of human monkeypox occurred from 1970 to 1979 in five Central and West African countries; 38 of these cases have been reported in Zaire. Children below 10 years of age comprised 83% of the cases. All cases have occurred in tropical rainforest areas, and clustering of cases has been observed in certain zones within countries and families [7].

In the 1980s, compared to the 1970s, a nine-fold increase in the number of confirmed and probable monkeypox cases was observed in the DRC (n = 343). In addition, 14 other cases were spread among four other African countries. Cases continued to increase in the 1990s, with 511 confirmed, probable, and/or possible monkeypox cases reported in the DRC and nine confirmed cases in Gabon. Between 2000 and 2009, monkeypox cases were reported in three African countries (DRC, Republic of the Congo, and South Sudan). Between 2010 and 2019, cases were found in seven African countries (Cameroon, the Central African Republic (CAR), the DRC, Liberia, Nigeria, Sierra Leone, and the Republic of the Congo). Compared to the last three decades of the 20th century, outbreaks in the year 2000 were greater due to the total number of cases and fewer in singular case reports [8]. There has been an increase in the number of individual outbreak reports over time since 1970. A total of 35 individual outbreaks have been reported outside the DRC, 20 of which have occurred since 2010 [9].

Notably, there has been limited viral spread to Europe and North America [8]. On May 18, 2022, 14, seven, and 13 cases of MPXV infection were reported in Portugal, Spain, and Canada, respectively. On May 19, 2022, Belgium, Sweden, and Italy confirmed their first MPXV cases. On May 20, 2022, Australia reported two cases; both patients recently returned from Europe. France, Germany, and the Netherlands confirmed their first cases on May 20, 2022, as well. The health secretary of the United Kingdom (UK) reported another 11 cases of MPXV on May 20, 2022, resulting in a total of 71 cases. Switzerland and Israel confirmed their first cases on May 21, 2022. Spain reported the first case on May 18, 2022. On June 3, 2022, Spain reported an increase of 20 cases, bringing the country's total cases to 186. On May 23, 2022, Denmark reported its first case. In Canada, Quebec announced 15 confirmed cases on May 24, 2022; the Czech Republic confirmed its first case on the same day. The United Arab Emirates confirmed its first case in late May 2022. Slovenia also confirmed its first case. By May 24, 2022, 19 countries had reported MPV cases.

The WHO issued a warning that the world may yet face another major challenge with a monkeypox outbreak after having met the challenges of the COVID-19 pandemic. Until June 10, 2022, 1,475 cases had been confirmed worldwide. The UK itself has reported 366 cases of monkeypox. In other countries like Spain (n = 275), Portugal (n = 209), Canada (n = 112), and the USA (n = 49), the number of cases has grown substantially. Therefore, this MPXV outbreak is becoming a concern for the whole world at present [1].

The incubation period of the MPXV ranges from five to 21 days, and the appearance and duration of symptoms and signs range from two to five weeks. The clinical pictures of this disease begin with nonspecific features such as fever, headaches, chills, asthenia, lethargy, lymph node swelling, back pain, and myalgia (muscle ache). Fever begins before rashes appear within a day to five days. After the fever, rashes of varying sizes appear on the face first and then appear on the whole body, including hands, legs, and feet [2].

Human MPXV infection is manifested by fever, headache, lymphadenopathy, muscle pain, and skin rash [10]. Rashes are centrifugal maculopapular rashes. The rash can be cutaneous or mucosal in the oral or anogenital region [11,12]. Respiratory distress with oral lesions, tonsillitis, difficulty swallowing, and epiglottitis affecting breathing also occur [13]. Skin lesions develop from macules, papules, vesicles, and pustules that become crusts and fall off [14].

The severity of the disease in infected patients depends on the host's immune competency and the titter of the genotype of the invading virus [15], with invasive modes of exposure causing more severe disease and a shorter incubation period [16]. Sporadic fatal cases have been reported, with fatalities ranging from 0% to 11% [17].

Smallpox vaccinations previously provided coincidental immunity to MPXV. The ACAM2000, a live-attenuated replicating vaccine, and JYNNEOS, a live-attenuated, non-replicating vaccine, are two US FDA-approved vaccines that can prevent monkeypox. However, ACAM2000 may cause serious side effects, including cardiac problems, whereas JYNNEOS is associated with fewer complications [18].

Recently, there has been an increase in global concern about monkeypox disease around the world [19]. Due to outbreaks in the West (in the USA, UK, and Ireland) along with the periodic re-emergence of the disease in parts of Africa, there’s increasing concern among global health bodies [20]. Since the first reporting of the MPXV infection in 2022 until April 13, 2023, a total of 86,956 cases from 110 countries have been reported, with 89 deaths (0.1% case fatality). Many countries reported a weekly increase, with the highest increase reported in Mexico (n = 72). In Saudi Arabia, eight laboratory-confirmed cases were reported [14], which resulted in panic regarding the spread of infection to more people across the country, especially during mass gatherings for Umrah and pilgrimage. We hypothesized that there would be anxiety among Saudi nationals and residents. In addition, there are not sufficient studies about anxiety from MPXV disease among the Saudi community. In this study, we aim to assess the anxiety and knowledge regarding monkeypox infection among the Saudi population and their willingness to take vaccines if available.

## Materials and methods

Ethical approval

The authors assert that all procedures contributing to this work comply with the ethical standards of the relevant national and institutional committees on human experimentation and with the Helsinki Declaration of 1975, as revised in 2008. This study received ethical approval from the Scientific Research Ethics Committee of Taif University (approval no. HAO-02-T-105). Filling out the questionnaire was considered consent or approval for participation in the study.

Study design and subjects

The intended cross-sectional study was designed to screen the knowledge about MPXV and the level of anxiety associated with getting ill among the Saudi population. A free online sample size calculator tool (Raosoft Inc., Seattle, WA, USA) was used to calculate the sample size with 99% confidence and a 2% margin of error. The inclusion criteria were adult Saudi citizens ≥ 18 years old, while children and adolescents below 18 years old were the only exclusion criteria from participation in the study. The study extended from September 1, 2022, to May 30, 2023.

Questionnaire design

The questionnaire included four sections. The questionnaire included demographic data such as age, sex, education, and nationality. It focused on disease knowledge with optimized questions of the standard generalized anxiety disorder 7 (GAD-7) assessment [21], as well as questions related to acceptance of the vaccine if made available. The questionnaire included knowledge questions about the signs of MPXV (skin rash and respiratory signs), MPXV transmission by close contact during sexual transmission, through skin lesions or saliva, and respiratory secretions from infected persons. It also included one question about the probability of MPXV having fatal consequences in some cases.

The GAD-7 questions were as follows: i) Have you ever had a panic attack (sudden, intense fear) in a manner that prevented you from carrying out your daily activities due to the probability of being infected with the monkeypox virus? ii) Have you been very afraid, even for a short time, of being infected with the monkeypox virus? iii) Has the constant thought of monkeypox bothered you so much that you couldn't get it out of your head or stop thinking about it? iv) Are you worrying too much, and do you feel that you have to wash your hands or use sanitizer over and over again? v) Do you find it difficult to relax from the thought of getting infected with the monkeypox virus? vi) Have you ever felt so anxious that it was difficult to sit still at the thought of being infected with the monkeypox virus? vii) Are you unable to stop or control your anxiety at the thought of contracting a monkeypox infection? The anxiety score was calculated using an online calculation tool [22]. If participants answered not at all, then the scores were 0, 1, 2, and 3 for not at all, several days, more than half the days, and nearly every day, respectively. The sum of scores was calculated: 0 to 4 is considered normal, while scores of 5 to 9, 10 to 14, and >15 were considered mild, moderate, and severe, respectively.

Statistical analysis

Data were analyzed using SPSS Statistics version 16.1 (IBM Corp., Armonk, NY, USA). Descriptive analysis with crosstabs was used to determine the frequencies and percentages. Different responses between groups based on anxiety level, age, sex, and educational level were evaluated using the chi-squared test and Spearman's correlation.

## Results

Effect of sex, age, and education on the development of anxiety toward infection by MPXV

There were a total of 5298 participants: 3109 (58.7%) males and 2189 (41.3%) females. Among the 5298 participants, 927 (17.5%) showed different degrees of GAD-7 anxiety towards MPXV infection: 832 (15.7%), 75 (1.4%), and 20 (0.4%) showed mild, moderate, and severe scores, respectively (Table 1). Females showed a significantly (P<0.001) higher rate of anxiety (487/2189, 22.2%) than males (440/3109, 14.2%). Most females and males showed a mild level of anxiety, and only small percentages showed a moderate (1.5% for females, 1.4% for males) or severe level of anxiety (0.3% for females, 0.5% for males). People aged 46 to 55 and >55 years showed significantly (P<0.001) higher rates of anxiety (30.7% and 27.2%) in comparison to other age groups (Table 1). Participants who studied medical sciences or were postgraduates showed a significantly lower level of anxiety (P<0.001) in comparison to other participants who are studying at the university, hold a bachelor’s degree, or have a secondary level of education (Table 1).

**Table 1:** The effects of the age and sex of the Saudi population on their knowledge and awareness of MPXV infection Values are expressed as numbers (%) ^1^ Healthcare includes physicians, nurses, and medical students ^2^ Bachelor includes people who hold a bachelor’s degree or are currently students in the university MPXV: Monkeypox virus, GAD-7: Generalized anxiety disorder scale-7

Variables		GAD-7					Cumulative	P and R values
		None	Mild	Moderate	Severe	Total affected		
		(0-4)	(5-9)	(10-14)	(15 and more)			
Sex	Male	2669 (85.8)	383 (12.3)	43 (1.4)	14 (0.5)	440 (14.2)	3109 (58.7)	P<0.001, R= 0.1
	Female	1702 (77.8)	449 (20.5)	32 (1.5)	6 (0.3)	487 (22.2)	2189 (41.3)	
Age (in years)	18-25	3273 (84.4)	535 (13.8)	56 (1.4)	14 (0.4)	605 (15.6)	3878(73.2)	P<0.001, R=0.09
	26-35	512 (81.5)	102 (16.2)	11 (1.8)	3 (0.5)	116 (18.5)	628 (11.9)	
	36-45	320 (78.8)	80 (19.7)	5 (1.2)	1 (0.2)	86 (21.2)	406 (7.7)	
	46-55	194 (68.6)	87 (30.7)	1 (0.4)	1 (0.4)	89 (31.4)	283 (5.3)	
	>55	72 (69.9)	28 (27.2)	2 (1.9)	1 (1.0)	31 (30.1)	103 (1.9)	
Education	Healthcare^1^	1035 (87.0)	142 (11.9)	10 (0.8)	3 (0.3)	155 (13.0)	1190 (22.5)	P<0.001, R=0.07
	Postgraduates	170 (88.5)	18 (9.4)	4 (2.1)	0 (0.0)	22 (11.5)	192 (3.6)	
	Bachelor^2^	1815 (82.6)	356 (16.2)	19 (0.9)	8 (0.4)	384 (17.4)	2198 (41.5)	
	Secondary school	1351 (78.6)	316 (18.4)	42 (2.4)	9 (0.9)	367 (21.4)	1718 (32.4)	
	Cumulative	4371 (82.5)	832 (15.7)	75 (1.4)	20 (0.4)	927 (17.5)	5298 (100)	

Effect of anxiety on the level of knowledge about MPXV

The responses of the participants with different degrees of anxiety showed a significant number of correct answers about how MPXV can lead to skin rash, transmission via contact with skin lesions, saliva, and upper respiratory secretions in infected people, and how MPXV can lead to fatal consequences in some cases. Among the 927 subjects who suffered from anxiety, significantly high numbers (P<0.001) know that MPXV can cause skin rash (704, 75.9%). They also know that the virus can be transmitted by contact with skin lesions, saliva, or respiratory secretions from infected patients (628, 67.7%) and can lead to fatal consequences (680, 73.4%). However, no significant variations were found between the people who did not show anxiety in comparison to people who showed different degrees of anxiety when asked about the ability of MPXV to cause respiratory distress or its transmission through sexual intercourse (Table 2). There was a positive correlation (0.103) between the anxiety level and the response of people toward the MPXV vaccine if it were available. A significant number (P<0.001) of the participants (617/832, 74.2%) showed mild anxiety, and 56/75 (74.7%) showed moderate anxiety and revealed their intention of getting the vaccine (Table 2). Meanwhile, a significantly higher reluctance to get the MPXV vaccine was recorded in people who did not show signs of anxiety (P<0.001) (Table 2).

**Table 2:** The awareness levels of Saudi nationals towards MPXV based on their GAD-7 score Values are expressed as numbers (%) Both chi-square and Spearman's correlation were used to measure the difference between different groups. MPXV: Monkeypox virus, GAD-7: Generalized anxiety disorder scale-7

Variables		GAD-7					Total	P and R values
									None	Mild	Moderate	Severe	Total affected
		(0-4)	(5-9)	(10-14)	(15 and more)								
		MPXV causes skin rash	No	118 (2.7)	24 (2.9)	11 (14.7)			2 (10.0)	37 (4.0)	155 (2.9)	P<0.001, R= -0.062
Yes	3086 (70.6)		648 (77.9)	46 (61.3)	10 (50.0)	704 (75.9)	3790 (71.5)					
Not sure	1167 (26.7)		160 (19.2)	18 (24.0)	8 (40.0)	186 (20.1)	1353 (25.5)					
MPXV causes respiratory signs	No	299 (6.8)	60 (7.2)	8 (10.7)	3 (15.0)	71 (7.7)	370 (7.0)	P<0.104, R=-0.037
	Yes	2488 (56.9)	506 (60.8)	46 (61.3)	11 (55.0)	563 (60.7)	3051 (57.6)	
	Not sure	1584 (36.2)	266 (32.0)	21 (28.0)	6 (30.0)	293 (31.6)	1877 (35.4)	
MPXV is transmitted by contact with skin lesions, saliva, and upper respiratory secretions of an infected person	No	352 (8.1)	61 (7.3)	10 (0.2)	3 (0.7)	74 (8.0)	426 (8.0)	P<0.001, R=-0.068
	Yes	2523 (57.7)	577 (69.4)	39 (52.0)	12 (60.0)	628 (67.7)	3151 (59.5)	
	Not sure	1496 (34.2)	194 (23.3)	26 (34.7)	5 (25.0)	225 (24.3)	1721 (32.5)	
MPXV is transmitted by close contact during sexual intercourse	No	436 (10.0)	83 (10.0)	10 (13.3)	3 (15.0)	96 (10.4)	532 (10.0)	P<0.685, R=0.018
	Yes	2072 (47.4)	408 (49.0)	38 (50.7)	11 (55.5)	457 (49.3)	2529 (47.7)	
	Not sure	1863 (42.6)	341 (41.0)	27 (36.0)	6 (30.0)	374 (40.3)	2237 (42.2)	
MPXV could be fatal in some cases	No	371 (8.5)	56 (6.7)	6 (8.0)	5 (25.0)	67 (7.2)	438 (8.3)	P<0.001, R=-0.066
	Yes	2405 (55.0)	518 (62.3)	52 (69.3)	13 (65.0)	583 (62.9)	2988 (56.4)	
	Not sure	1595 (36.5)	258 (31.0)	17 (22.7)	2 (10.0)	277 (29.9)	1872 (35.3)	
If the MPXV vaccine is available, do you intend to take it?	No	1925 (44.0)	215 (25.8)	19 (25.3)	13 (65.0)	247 (26.6)	2172 (41.0)	P<0.001, R=0.103
	Yes	2446 (56.0)	617 (74.2)	56 (74.7)	7 (35.0)	680 (73.4)	3126 (59.0)	
	Cumulative	4371 (82.5)	832 (15.7)	75 (1.4)	20 (0.4)	927 (17.5)	5298 (100)	

Effect of age and sex of the Saudi population on knowledge and awareness of MPXV infection

Younger age groups seem to possess relatively more knowledge about the disease than older age groups. However, participants with an age range of 36 to 45 years showed the highest correct response (79.3%) when asked about skin rash caused by MPXV, while participants >55 years showed the lowest correct answer (60.2%). The participants with an age range of 36 to 45 years also showed the highest correct response (65.8%) when asked if MPXV can be transmitted by skin lesions, saliva, or respiratory secretions (P>0.001) (Table 3). A low percentage of participants with ages ranging from 46 to 55 (36%) and >55 years (34%) know that MPXV can be transmitted by close contact during sexual intercourse in comparison to other age groups (Table 3). There was no significant variation in the acceptance of getting the vaccine among the age groups (Table 3). Females showed significantly higher knowledge than males regarding MPXV induction of skin rashes, its respiratory signs, and transmission via skin lesions or contact with saliva or respiratory secretions from infected persons (P<0.001). They were found to possess a higher acceptance rate of vaccination whenever feasible (64.7% in females vs. 55% in males). However, males were significantly more oriented (54.3%) than females (38.4%) when asked about the sexual transmission of MPXV (P<0.001) (Table 3).

**Table 3:** Effects of the age and sex of the Saudi population on the knowledge and awareness of MPXV infection Values are expressed as numbers and (%) ^1^P <0.001, R = -0.049 for sex, -0.028 for age ^2^ P<0.001, R = -0.030 for sex, -0.042 for age ^3^ P<0.001, for sex, and 0.14 for age. R = -0.030 for sex, 0.006 for age ^4^ P<0.001, R = -0.093 for sex, 0.013 for age ^5^ P<0.001, R = -0.030 for sex, -0.094 for age ^6^ P<0.001, R = -0.097 for sex, P<0.0603, R = -0.019 for age MPXV: Monkeypox virus

Variable		Sex		Age (in years)					Total
		Male	Female	18-25	26-35	36-45	46-55	> 55	
MPXV causes skin rash^1^	No	98 (3.2)	57 (2.6)	110 (2.8)	26 (4.1)	9 (2.2)	5 (1.8)	5 (4.9)	155 (2.9)
	Yes	2152 (69.2)	1638 (74.8)	2746 (70.8)	452 (72.0)	322 (79.3)	208 (73.5)	62 (60.2)	3790 (71.5)
	Not sure	859 (27.6)	494 (22.6)	1022 (26.4)	150 (23.9)	75 (18.5)	70 (24.7)	36 (35.0)	1353 (25.5)
MPXV causes respiratory signs^2^	No	259 (8.3)	110 (5.0)	244 (6.3)	56 (8.9)	37 (9.1)	19 (6.7)	13 (12.6)	369 (7.0)
	Yes	1659 (53.4)	1392 (63.6)	2312 (59.6)	347 (55.3)	211 (52.0)	133 (47.0)	48 (46.6)	3051 (57.6)
	Not sure	1190 (38.3)	687 (31.4)	1321 (34.1)	225 (35.8)	158 (38.9)	131 (46.3)	42 (40.8)	1877 (35.4)
MPXV is transmitted by contact with skin lesions, saliva, and upper respiratory secretions of an infected person^3^	No	299 (9.6)	127 (5.8)	318 (8.2)	58 (9.2)	22 (5.4)	22 (7.8)	6 (5.8)	426 (8.0)
	Yes	1721 (55.4)	1430 (65.3)	2304 (59.4)	352 (56.1)	267 (65.8)	165 (58.3)	63 (61.2)	3151 (59.5)
	Not sure	1089 (35.0)	632 (28.9)	1256 (32.4)	218 (34.7)	117 (28.8)	96 (33.9)	34 (33.0)	1721 (32.5)
MPXV is transmitted by close contact during sexual intercourse^4^	No	274 (8.8)	258 (11.8)	381 (9.8)	68 (10.8)	37 (9.1)	31 (11.0)	15 (14.6)	532 (10.0)
	Yes	1688 (54.3)	841 (38.4)	1872 (48.3)	319 (50.8)	201 (49.5)	102 (36.0)	35 (34.0)	2529 (47.7)
	Not sure	1147 (36.9)	1090 (49.8)	1625 (41.9)	241 (38.4)	168 (41.4)	150 (53.0)	53 (51.6)	2237 (42.2)
MPXV could be fatal in some cases^5^	No	304 (9.8)	134 (6.1)	293 (7.6)	62 (9.9)	50 (12.3)	24 (8.5)	9 (8.7)	438 (8.3)
	Yes	1753 (56.4)	1235 (56.4)	2313 (59.6)	319 (50.8)	192 (47.3)	115 (40.6)	49 (47.6)	2988 (56.4)
	Not sure	1052 (33.8)	820 (37.5)	1272 (32.8)	247 (39.3)	164 (40.4)	144 (50.9)	45 (43.7)	1872 (35.3)
If the MPXV vaccine is available, do you intend to take it?^ 6^	No	1399 (45.0)	773 (35.3)	1566 (40.4)	272 (43.3)	174 (42.9)	116 (41.0)	44 (42.7)	2172 (41.0)
	Yes	1710 (55.0)	1416 (64.7)	2312 (59.6)	356 (56.7)	232 (57.1)	167 (59.0)	59 (57.3)	3126 (59)
	Cumulative	3109 (58.7)	2189 (41.3)	3878 (73.2)	628 (11.9)	406 (7.7)	283 (5.3)	103 (1.9)	5298 (100)

Effects of the level of education on knowledge and awareness of MPXV infection

There was no significant difference among the different educational backgrounds of the participants when asked two questions regarding the sexual transmission of MPXV or whether it can cause respiratory distress. In addition, there was no significant variation in vaccine acceptance among participants with different educational levels (Table 4). Significant variations were detected in response to the questions asking about the probability of skin rash in MPXV infection as well as the possible fatal consequence in some cases in addition to the transmission of MPXV by contact with a skin lesion, saliva, or respiratory secretions. Although we hypothesized that people with medical backgrounds are assumed to have better knowledge and a higher rate of vaccine acceptance than participants with different educational backgrounds, the participants with medical backgrounds did not show any significant variations except for two knowledge questions only, i.e., the questions on MPXV-induced rash and possible MPXV fatal consequences (Table 4).

**Table 4:** Level of education of the Saudi population and its impact on the knowledge and awareness of MPXV infection MPXV: Monkeypox virus

Variables		Education				Total	P and R values
		Healthcare	Postgraduate	Bachelor	Secondary		
MPXV causes skin rash	No	19 (1.6)	7 (3.6)	58 (2.6)	71 (4.1)	155 (2.9)	P<0.005, R= -0.018
	Yes	868 (72.9)	130 (67.7)	1570 (71.4)	1222 (71.1)	3790 (71.5)	
	Not sure	303 (25.5)	55 (28.6)	570 (25.9)	425 (24.7)	1353 (25.5)	
MPXV causes respiratory signs	No	69 (5.8)	16 (8.3)	141 (6.4)	143 (8.3)	369 (7.0)	P<0.089, R = -0.026
	Yes	690 (58.0)	103 (53.6)	1264 (57.5)	994 (57.9)	3051 (57.6)	
	Not sure	430 (36.3)	73 (38.0)	793 (36.1)	581 (33.8)	1877 (35.4)	
MPXV is transmitted by contact with skin lesions, saliva, and upper respiratory secretions of an infected person	No	87 (7.3)	165 (7.5)	155 (9.0)	19 (9.9)	426 (8.0)	P<0.001, R = -0.058
	Yes	786 (66.1)	1274 (58.0)	1082 (63.0)	109 (56.8)	3151 (59.5)	
	Not sure	317 (26.6)	759 (34.5)	481 (28.0)	64 (33.3)	1721 (32.5)	
MPXV is transmitted by close contact during sexual intercourse	No	123 (10.3)	25 (13.0)	200 (9.1)	184 (10.7)	532 (10.0)	P<0.133, R = -0.018
	Yes	573 (48.2)	99 (51.6)	1031 (46.9)	826 (48.1)	2529 (47.7)	
	Not sure	494 (41.5)	68 (35.4)	967 (44.0)	708 (41.2)	2237 (42.2)	
MPXV could be fatal in some cases	No	84 (7.0)	27 (14.1)	166 (7.6)	161 (9.4)	438 (8.3)	P<0.001, R = -0.025
	Yes	690 (58.0)	100 (52.1)	1157 (52.6)	1041 (60,6)	2988 (56.4)	
	Not sure	416 (350)	65 (33.8)	875 (39.8)	516 (30.0)	1872 (35.3)	
If the MPXV vaccine is available, do you intend to take it?	No	471 (39.5)	91 (47.4)	914 (41.6)	696 (40.5)	2172 (41.0)	P<0.188, R = -0.013
	Yes	719 (60.5)	101 (52.6)	1284 (58.4)	1022 (59.5)	3126 (59.0)	
Total		1190 (22.5)	192 (3.6)	2198 (41.5)	1718 (32.4)	5298 (100)	

## Discussion

Anxiety is considered one of the most common mental disorders. It affects 20% of adult humans in the U.S. [23]. In the current study, females were more likely to have anxiety disorder (22.2%) in comparison to males (14.2%). This finding matches one of the most common national surveys, which revealed an anxiety score of 30.5% for females vs. 19.2% for males [24]. It is also well-matched by other well-documented psychiatric epidemiology studies [25-27].

It is well known that GAD disorders, which manifest through excessive prediction of negative impacts of events, are lower in older than younger age groups [28]. It is probably a true age-related syndrome that attenuates over time or maybe because of the adoption of less relevant diagnostic criteria for older adults [29]. The lifetime prevalence of GAD among older adults is 3.6% in those aged 60 and older [30] and 1.2% in those aged 65 and older [31]. However, in the current study, older participants (46 to 55 and >55 years) showed higher percentages of GAD in comparison to younger participants (18 to 45 years). This could be explained by the fact that older adults showed specific worry about issues related to their health and diseases that may affect them [32], while younger adults showed less worry about other issues that are of major concern to them, including their performance in education, income, work, and social relationships [33].

Inconsistent results were reported about the relationship between anxiety and education level [34-37]. Only one study [36] reported a clear inverse association between educational level and depression. The only longitudinal study that addressed education as the main predictor of depression reported that the association was weakened and no longer statistically significant when adjusted for earlier depressive symptoms [35]. These results imply no cumulative effect of education on depression with age. A study not included in the meta-analysis that followed adolescents into early adulthood did not show an association between educational attainment and depression at all [38].

The current study reveals a gap in knowledge about MPXV, with high numbers that did not give correct answers to the questions regarding signs, transmission, and possible fatalities from the disease. This could be attributed to the lack of intensive activities to raise awareness among the Saudi population, as the frequency of the disease was not prominent at the time of this survey. The responses in the current study were similar to those reported in a recent study from Saudi Arabia [39] and Jordan [40], but not to those reported among the Chinese population [41].

Although statistically significant, we noticed that people with medical backgrounds did not show satisfactory knowledge levels about MPXV in comparison to other groups. This finding may need further attention, especially while adopting control and prevention efforts for MPXV. It is known that healthcare workers with good knowledge are important to the proper adoption of primary preventive measures by patients [42,43].

Currently, there is no homologous vaccine used for MPXV; however, approved orthopoxvirus cross-reacts with MPXV and could be effectively used against MPXV infection [44,45]. Pre- and post-exposure prophylaxis can be adopted to protect against MPXV infection. Both ACAM2000 and JYNNEOS, the smallpox vaccines, are approved in the USA to protect against MPXV [44,45].

Limitations

The study included participants who were recruited using social media, as we could not reach people who were not on these platforms. In addition, the age range of the majority of the participants (73.2%) was 18 to 25 years, which may not represent the total community.

## Conclusions

A relatively high percentage of Saudis show a moderate level of anxiety about MPXV. Awareness about the disease is needed since there is a low level of knowledge among Saudi nationals. A considerable percentage of the participants (59.0%) showed acceptance of the MPXV vaccine if it were made available. With major mass gathering events such as the Umrah and pilgrimage, strict precautions should be considered to ensure that people are free from MPXV before traveling across Saudi Arabia. It is suggested that screening tests be conducted for travelers before entering the country.
